# Gain-of-function mutation in *SCN11A* causes itch and affects neurogenic inflammation and muscle function in *Scn11a*^*+/L799P*^ mice

**DOI:** 10.1371/journal.pone.0237101

**Published:** 2020-08-20

**Authors:** Matthias Ebbinghaus, Lorena Tuchscherr, Gisela Segond von Banchet, Lutz Liebmann, Volker Adams, Mieczyslaw Gajda, Christian A. Hübner, Ingo Kurth, Hans-Georg Schaible

**Affiliations:** 1 Institute of Physiology 1/Neurophysiology, University Hospital - Friedrich Schiller University Jena, Jena, Germany; 2 Institute of Medical Microbiology, University Hospital - Friedrich Schiller University Jena, Jena, Germany; 3 Institute of Human Genetics, University Hospital - Friedrich Schiller University Jena, Jena, Germany; 4 Laboratory of Molecular and Experimental Cardiology, Heart Center Dresden, Technische Universität Dresden, Dresden, Germany; 5 Institute of Pathology, University Hospital - Friedrich Schiller University Jena, Jena, Germany; 6 Institute of Human Genetics, Medical Faculty - RWTH Aachen University, Aachen, Germany; University Hospital Wurzburg, GERMANY

## Abstract

Mutations in the genes encoding for voltage-gated sodium channels cause profound sensory disturbances and other symptoms dependent on the distribution of a particular channel subtype in different organs. Humans with the gain-of-function mutation *p*.*Leu811Pro* in *SCN11A* (encoding for the voltage-gated Na_v_1.9 channel) exhibit congenital insensitivity to pain, pruritus, self-inflicted injuries, slow healing wounds, muscle weakness, Charcot-like arthropathies, and intestinal dysmotility. As already shown, knock-in mice (*Scn11a*^*+/L799P*^) carrying the orthologous mutation *p*.*Leu799Pro* replicate reduced pain sensitivity and show frequent tissue lesions. In the present study we explored whether *Scn11a*^*+/L799P*^ mice develop also pruritus, muscle weakness, and changes in gastrointestinal transit time. Furthermore, we analyzed morphological and functional differences in nerves, skeletal muscle, joints and small intestine from *Scn11a*^*+/L799P*^ and *Scn11a*^*+/+*^ wild type mice. Compared to *Scn11a*^*+/+*^ mice, *Scn11a*^*+/L799P*^ mice showed enhanced scratching bouts before skin lesions developed, indicating pruritus. *Scn11a*^*+/L799P*^ mice exhibited reduced grip strength, but no disturbances in motor coordination. Skeletal muscle fiber types and joint architecture were unaltered in *Scn11a*^*+/L799P*^ mice. Their gastrointestinal transit time was unaltered. The small intestine from *Scn11a*^*+/L799P*^ showed a small shift towards less frequent peristaltic movements. Similar proportions of lumbar dorsal root ganglion neurons from *Scn11a*^*+/L799P*^ and *Scn11a*^*+/+*^ mice were calcitonin gene-related peptide (CGRP-) positive, but isolated sciatic nerves from *Scn11a*^*+/L799P*^ mice exhibited a significant reduction of the capsaicin-evoked release of CGRP indicating reduced neurogenic inflammation. These data indicate important Na_v_1.9 channel functions in several organs in both humans and mice. They support the pathophysiological relevance of increased basal activity of Na_v_1.9 channels for sensory abnormalities (pain and itch) and suggest resulting malfunctions of the motor system and of the gastrointestinal tract. *Scn11a*^*+/L799P*^ mice are suitable to investigate the role of Na_v_1.9, and to explore the pathophysiological changes and mechanisms which develop as a consequence of Na_v_1.9 hyperactivity.

## Introduction

Essential body functions depend on ion channels such as voltage-gated sodium channels (VGSC). VGSC produce action potentials in neurons, and regulate neuronal excitability. Since there are several VGSC isoforms (Na_V_1.1-Na_V_1.9) the functional significance of each isoform needs to be established. Important contributions to the understanding of VGSC functions are provided by the detection of genetic variants of VGSC and their related dysfunctions in humans (channelopathies) [1, 2]. Leipold et al. and Woods et al. identified in three young individuals a gain-of-function mutation (*p*.*Leu811Pro*) in *SCN11A*, the gene that encodes for Na_V_1.9 [3, 4]. A corresponding case was reported by Salvatierra et al. [5]. Na_V_1.9 is preferentially expressed in dorsal root ganglion (DRG) neurons (including functionally identified nociceptors), trigeminal neurons and intrinsic myenteric neurons [6]. Na_V_1.9 has also been reported in hair cells of the auditory and vestibular system, in the retina and in layer V of the medial prefrontal cortex [7]. In general, Na_V_1.9 acts as a threshold channel by contributing a sodium conductance that regulates resting potentials and prolongs the depolarizing response to subthreshold stimuli [6].

Humans with the gain-of-function mutation *p*.*Leu811Pro* in *SCN11A* (detected by Leipold et al. and Woods et al. in unrelated patients, 8–13 years old) show congenital insensitivity to pain, pruritus, self-inflicted injuries, slow healing wounds, intestinal dysmotility, mild muscle weakness, Charcot-like arthropathies and further disturbances [3, 4] suggesting that Na_V_1.9 is functionally important in several organs. The patient reported by Salvatierra et al. [5] displayed similar symptoms, in particular severe pruritus, a partial loss-of pain sensation, fractures with little trauma, constipation and intermittent diarrhea, heterotrophic ossification, and some others. The gain-of-function mutation *p*.*Leu811Pro* in *SCN11A* was observed in female and male patients [3–5]. In order to characterize the biophysics of this channelopathy, knock-in mice (*Scn11a*^*+/L799P*^) carrying the orthologous mutation *p*.*Leu799Pro* were generated [3, 5]. Such mice also allow to investigate the importance of Na_V_1.9 in different body functions and to explore whether Na_V_1.9 is similarly involved across different species. In biophysical experiments it was shown that the *p*.*Leu811Pro* alteration increases the basal activity of Na_v_1.9 channels, causing an excess sodium ion influx at rest and subsequent cell depolarization [3]. As a consequence, other voltage-gated sodium channels such as Na_v_1.7, Na_v_1.8 and voltage-gated calcium ion channels (they form the main constituents of the action potential in DRG neurons) may undergo progressive inactivation, resulting in impaired generation of action potentials [3].

*Scn11a*^*+/L799P*^ mice frequently show tissue lesions, and in tail-flick experiments they exhibit a significantly higher threshold for noxious heat stimuli than wild-type littermates [3]. Under basal conditions, the withdrawal threshold for plantar mechanical and thermal stimulation did not differ between genotypes, but after zymosan-induced inflammation *Scn11a*^*+/L799P*^ mice showed a smaller reduction in the withdrawal threshold and less protective behavior (guarding), indicating less thermal and mechanical hyperalgesia [3]. Since the excess sodium ion influx at rest and subsequent cell depolarization may cause progressive inactivation (see above), impaired generation of action potentials may explain why pain perception is impaired although the activation of the normal Na_v_1.9 channel is rather involved in increased pain perception (hyperexcitability) during inflammation [7].

The present study had several aims. First, we explored whether *Scn11a*^*+/L799P*^ mice exhibit pruritus, changes in motor performance, changes in skeletal muscles and joints, and disturbances in intestinal dysmotility. Such symptoms would indicate an involvement of Na_v_1.9 in organic functions in mice similar as in humans, and they would indicate that excess sodium ion influx through Na_v_1.9 is pathophysiologically relevant in different tissues. Second we studied whether nerves from *Scn11a*^*+/L799P*^ mice exhibit abnormalities in their secretory function. We quantified the proportion of calcitonin gene-related peptide- (CGRP-) positive DRG neurons, and measured the release of CGRP from peripheral nerves upon stimulation with capsaicin because CGRP was implicated in injury, neurogenic inflammation [8], wound healing [9–11], pain [12], and itch [13]. We found that *Scn11a*^*+/L799P*^ mice exhibit similar functional disturbances as humans with the *p*.*Leu799Pro* mutation suggesting comparable functional importance of Na_v_1.9 across species. Furthermore, we found a significant deficit of the sciatic nerve to release CGRP suggesting that the reduction of CGRP release may be involved in the pathophysiological consequences of the mutation.

## Materials and methods

### Animals

We used male/female heterozygous *Scn11a*^*+/L799P*^ knock-in mice and wild-type littermates (*Scn11a*^*+/+*^) with *C57BL/6J* background in this study. *Scn11a*^*+/L799P*^ mice were genotyped as previously described [3]. For genotyping we used primers adjacent to the loxP site (5´-gcagtccccatcaaaattcc-3´, within the *Scn11a* locus and 5´-gaatcgatcctagagaattccg-3´, sequence comes with the loxP site). The PCR reaction results in a 272 bp amplicon for the knockin allele. Homozygous *Scn11a*^*L799P/L799P*^ mice could not be bred in sufficient number. At the end of the experiments mice were killed by cervical dislocation under deep isoflurane (5.0%) anesthesia or in a CO_2_ atmosphere, respectively.

Mice were age-matched for each experiment but within the age group mice were not selected regarding sex. All tests were performed and analyzed with the experimenter blinded to genotype. All animals were bred and maintained under the same specific pathogen-free conditions by the Animal Facility of the University Hospital Jena. All experiments on animals were performed according to the Tierschutzgesetz der Bundesrepublik Deutschland and were approved by the Thuringian Government (Thüringer Landesamt für Verbraucherschutz). The animals were treated in accordance with the guiding principles in the care and use of animals. Data sampling, evaluation, and presentation complied with the ARRIVE guidelines.

### Quantitative analysis of scratching and grooming behavior

Both hindlimb scratching bouts and forelimb grooming chains were counted between 9 am and 3 pm as previously described [5, 14, 15]. Briefly, mice free of skin lesions were placed separately in the observation tubes (diameter: 9 cm; height: 20 cm) on a grid to get acclimatized for 20 min. Then, mice were video-recorded from a front view for 30 min. Afterwards the total number of scratching bouts and grooming chains were counted. A bout of scratching was defined as a continuous fast scratching, not wiping or stroking, movement of a hind paw directed toward the neck area. A bout ended when the mouse either licked its hind paw or placed it back on the grid. Single bouts are identified by a break of at least 5 sec. A grooming chain consists of 4 phases of wiping, stroking and body licking [15] and was counted respectively. Each mouse was tested three times. The first testing was performed in order to render the mouse familiar with the testing situation. After three and five days the mouse was tested again and the values were taken for analysis.

### Immunohistochemical labeling for CGRP in DRG sections

Dorsal root ganglia (DRG) from segments L1-5 from both sides were excised separately, fixed at 4°C in 4% paraformaldehyde for 24 hours, embedded in paraffin, cut into 5 μm sections which were dewaxed and autoclaved for 15 min (120°C, 1 bar). For CGRP labeling we applied overnight at 4°C a mouse reactive anti-CGRP antibody (1:100; polyclonal against a synthetic rat Tyr-CGRP(23–37) conjugated to gamma globulin developed in goat; Cat.No BP022, Acris antibodies, Herford, Germany; RRID:AB_973533). Sections were incubated for 2 hours in biotinylated rabbit anti-goat antibody (1:200; DAKO, Glostrup, Denmark), then the avidin-biotin peroxidase complex (Vectastatin-Elite ABC Kit, Vector Laboratories, Burlingame, USA) was applied for 40 min. Sections were developed with Jenchrom px blue (JenLab, Jena, Germany). The immunohistochemical labeling was evaluated as previously described [16]. In each second section the proportion of neuronal profiles with CGRP-like immunoreactivity (IR) was determined using a light microscope coupled to an image analyzing system (AxioVision, Zeiss). For each mouse the average proportion of labeled neuronal profiles was calculated. Per mouse at least 100 neuronal profiles with a visible nucleus were counted. In a control experiment the primary antibodies were omitted.

### Sciatic nerves preparation and measurement of CGRP release

Sciatic nerves were excised out of the lumbar plexus and dissected along the hind limb as described previously [17]. The epi-perineurium was widened under a binocular microscope, and then the nerves were washed separately in 1 ml carbogen-gassed (95% O_2_, 5% CO_2_) synthetic interstitial fluid (SIF, in mM: 107.8 NaCl, 3.5 KCl, 0.7 MgSO_4_, 26.2 NaHCO_3_, 1.7 NaH_2_PO_4_, 1.5 CaCl_2_, 9.6 sodium gluconate, 5.6 glucose, 7.6 sucrose; [18]), pH 7.4 at 32 °C, for 30 min. Thereafter the nerves were incubated for 5 min in 150 μl SIF in the first test tube to obtain a baseline, and then they were forwarded to the second test tube and so forth (all steps with a volume of 150 μl SIF at 32 °C). For stimulation we used capsaicin (Sigma-Aldrich, Taufkirchen, Germany), dissolved in ethanol and freshly diluted with SIF to 1 μM, 0.1 μM and 0.01 μM respectively. The samples were supplemented with 5-fold concentrated CGRP ELISA buffer (bertin pharma, Montigny-le-Bretonneux, France) and immediately stored at -20 °C. For CGRP measurement we used an enzyme immunoassay kit (bertin pharma) according to the manufacturer’s instructions.

### Gastrointestinal transit time

Mice were placed in single cages and were force-fed with a single bolus of 250 μl of a water solution containing 2 x 10^7^ colonies of strictly aerobic *Bacillus subtilis* strain DSM 618 (Merck Millipore, Burlington, Massachusetts) as an inert transit marker. All feces were collected immediately and after 3, 6, 9, 15 and 24 h and stored at -20° C. Afterwards feces were dried at 37° C for 24 h and aqueous slurries (40 mg/ml) were prepared [19]. Slurries were filtered by 250 μm tissue strainers (Thermo Fisher Scientific, Waltham, MA, USA) and serial dilutions of this suspension were made and spread on blood agar plates to obtain counts of 30–300 colonies per plate. Agar plates were incubated at 37° C for 20 h. *Bacillus subtilis* produced small white colonies that were verified by mass spectrometry analysis (Vitek-MS, Biomérieux, Marcy-l’Étoile, France) for the correct identification. Colony forming units (CFU) per ml were calculated for each single mouse and each time point. The mean transit time (MTT in h) was calculated as previously described [19], following the formula MTT = (Sum(m_i_ x t_i_)/Sum(m_i_)); m_i_ = number of spores present in the feces, t_i_ = time interval. The mean value was obtained from 2 independent series.

### Measurement of peristaltic movements

Spontaneous mechanical activity of the intestine was measured in isolated segments of the intestine of freshly sacrificed mice as described before [20]. Briefly, after decapitation, the duodenum from adult mice was immediately removed and placed in Krebs buffer composed of (in mM): 120 NaCl, 3.5 KCl, 1.3 MgSO_4_x7H_2_O, 1.25 NaH_2_PO_4_, 2 CaCl_2_x2H_2_O, 25 NaHCO_3_, 10 D-Glucose; gassed with 95% O_2_ and 5% CO_2_, pH 7.4. Short pieces (2–3 cm) of the intestine were prepared and transferred to an interface recording chamber and perfused with oxygenated Krebs solution (2–3 ml/min) at 34°C. Extracellular field potentials were recorded using glass microelectrodes (impedance 2–3 MΩ) from the Tunica muscularis. Electrodes were filled with Krebs solution. Data of field potential recordings were collected with an extracellular amplifier (EXT-02, NPI electronic, Tamm, Germany), low pass filtered at 4 kHz and digitally stored with a sample frequency of 10 kHz. Analysis of field potential recordings was performed using the software “Signal” (Cambridge Electronic Design, UK).

### Quantitative gait analysis

Initially, mice were trained to walk on a horizontal plastic beam (1000 mm long, 38 mm broad) leading to their home cage. After the learning phase one beam-walking trial was digital video-recorded from a rear view for each animal. The video sequences were analyzed to select single frames in which the mice were seen in phases of the gait cycle, meeting the criteria for measurements as described before [21]. Measurements of the foot-base-angle at toe-off positions of the hind-paws were performed with the ImageJ 1.50b program (Wayne Rasband, National Institutes of Health, USA). Each mouse was tested twice at two different days and one mean value was calculated for each mouse.

### Ladder-climbing test

Evaluation of skilled walking, indicating quantitative evaluation of complex motor behavior, was performed by the ladder-climbing test as described before [22]. The ladder (length: 100 cm; rung distance: 2 cm; rung thickness: 2 mm; rung width: 6 cm) was fixed in an inclined position (45°) and each single mouse was placed at the bottom rungs of the ladder. Climbing was video recorded from a front view. The numbers of correct steps (correct placing of the hind paw and sustained position until the next forward move) and of missteps from two trials were counted, evaluated qualitatively and the mean was displayed. Each mouse was tested three times at two different days and one mean value was calculated for each mouse.

### Grip strength

Measurement of grip strength was performed using single weights each consisting of a ball of tangled fine gauge stainless steel wire attached to a series of steel chain links as described before [23]. The weights are 2.8, 9.2, 15.4, 19, 34.2, 42.5, 50, 60.4, 73, 86.5 and 103 g. Each mouse is held by the middle of the tail and lowered to allow it to grasp the first weight which is lying on the laboratory bench with its forepaws. As it grasps the weight the mouse is raised until the weight is clear of the bench. If the mouse holds the weight two times for three seconds the next heaviest weight will be applied after a break of at least 15 min. If the mouse fails the trial is terminated and the maximum weight is documented. Each mouse was tested at two different days and the mean value of two testings was obtained from each mouse.

### Histological analysis of muscle and joints

Joint diameters were measured using an Oditest vernier caliper (Kroeplin, Schlüchtern, Germany). Muscle fiber typing was performed as described before [24]. Briefly, paraffin cross sections (4–8 μm) of the paraformaldehyde (4%) fixed forelimb and of the separated Musculus tibialis cranialis were incubated with the primary antibody (1:75, mouse monoclonal Anti-Slow Skeletal Myosin Heavy chain [ab11083]; Abcam, Cambridge, UK; PRID:AB_297734) for 1 h at room temperature. After washing the sections were incubated with the secondary antibody (1:250, rabbit polyclonal Anti-Mouse IgG-Peroxidase [A9044]; Sigma-Aldrich, Taufkirchen, Germany) for 45 min at room temperature. Specifically bound antibodies were detected using a CSA-II kit (Dako, Hamburg, Germany) and slow type specific fibers were counted under a microscope. Per mouse at least 500 fibers were counted.

For histopathology, paws were removed, fixed in 5.52% formalin, decalcified (IMMUNOCAL, quartett, Berlin, Germany), embedded in paraffin and cut into 2 μm frontal sections which were stained with hematoxylin and eosin (H&E).

### Statistical analysis

All data are expressed as means ± SEM unless otherwise stated. Differences between groups were calculated using the two-tailed Student´s t-test for unpaired and normally distributed values (for testing for normality we used the Kolmogorov-Smirnov test). Statistical significance was calculated with SPSS (v.16.0, Chicago, USA) software program, and accepted at p<0.05.

## Results

### Quantification of scratching behavior and grooming activity

**Fig 1:**
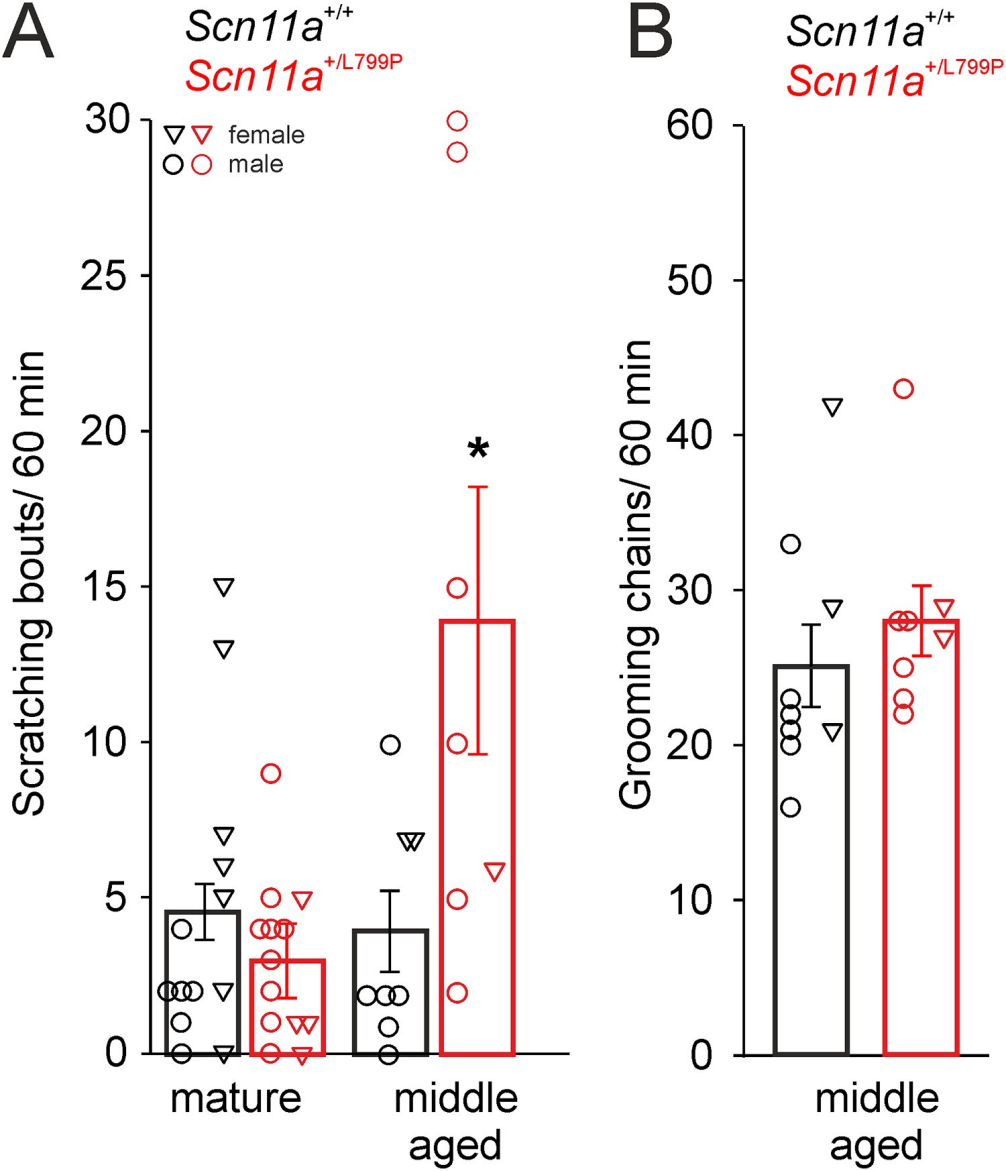
Quantification of scratching and grooming behavior of *Scn11a*^*+/+*^ and *Scn11a*^*+/L799P*^ mice. (A) Middle aged (9–12 months) but not mature adult (3–5 months) *Scn11a*^*+/L799P*^ mice showed enhanced numbers of scratching bouts (n = 7–13 per group). (B) No differences in the number of grooming chains were observed between both genotypes (n = 8–9 per group). Triangles indicate values of individual female mice, circles indicate values of individual male mice. Values are mean ± SEM. *p < 0.05.

We quantified the count of scratching bouts in a total time span of 60 min by video analysis and found in the total group of mice (not differentiating between male and female) a significantly increased number of bouts in older, middle aged (9–12 months [25]) *Scn11a*^*+/L799P*^ mice compared to their wild-type (*Scn11a*^*+/+*^) littermates. Younger, mature adult (3–5 months [25]) mice did not show any differences between both genotypes (Fig 1A). Notably all mice were free of skin lesions at the time point of observation. To verify that the increased frequency of scratching is not due to a generally altered behavioral disorder we also quantified the stereotype chains of grooming in middle aged mice. We did not find a significant difference in grooming activity between both genotypes (Fig 1B).

Triangles and circles in Fig 1 show the values of the individual animals and indicate whether the particular mouse was male or female. This retrospective analysis showed that at the middle age only 2 female mice were included in the analysis. Therefore it cannot be excluded that female mice could be different from male mice.

### Localization of CGRP-immunoreactivity in DRG neurons and release of CGRP from sciatic nerve axons

**Fig 2:**
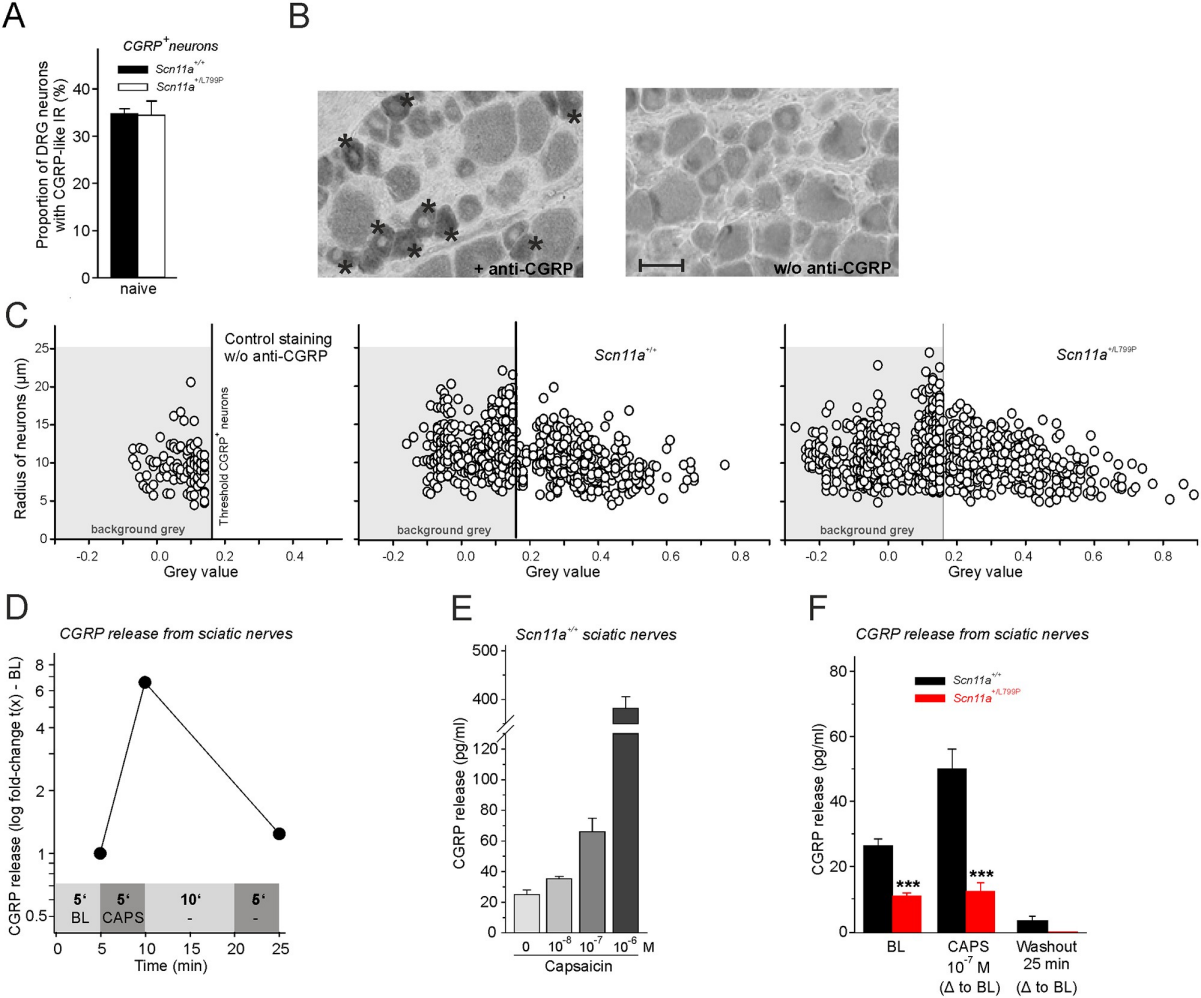
Localization of CGRP-immunoreactivity in lumbar DRG neurons and CGRP release from sciatic nerve axons of *Scn11a*^*+/+*^ and *Scn11a*^*+/L799P*^ mice. (A) Proportions of DRG neurons with CGRP-like immunoreactivity in lumbar sections from *Scn11a*^*+/+*^ and *Scn11a*^*+/L799P*^ mice (n = 5–7 mice per group). (B) Sections of lumbar DRGs. The left section contains CGRP-positive neurons (labeled by stars), the right section was processed without anti-CGRP antibody for control. (C) Display of all DRG neurons analyzed for CGRP-like immunoreactivity. Each neuron is defined by its radius and its grey value. Neurons in the left panel, not exposed to the anti CGRP antibody, show the background grey level. Neurons in the other panels were counted as CGRP-positive if they had grey values above background. (D) Protocol of CGRP release experiments from sciatic nerve axons. Values from one nerve of a *Scn11a*^*+/+*^ mouse with 10^−7^ M capsaicin are shown as an example. Areas in grey above the x-axis indicate the incubation time (in minutes) in the different test tubes. (E) CGRP release from sciatic nerve axons from naïve *Scn11a*^*+/+*^ mice without and after stimulation with different concentrations of capsaicin (n = 4–6 per concentration). (F) *Scn11a*^*+/L799P*^ mice (3–7 months old) showed significantly less CGRP release from sciatic nerve axons in baseline (n = 14–21 nerves per group) and after stimulation with capsaicin (n = 8–11 nerves per group). Values are mean ± SEM. ***p < 0.001. *BL* baseline, *CAPS* capsaicin, *CGRP* calcitonin gene-related peptide, Δ difference.

We determined the proportion of lumbar DRG neurons of middle aged mice (9–13 months old) which express CGRP-immunoreactivity. As shown in Fig 2A, the proportion of DRG neurons with CGRP-like immunoreactivity was almost identical in *Scn11a*^*+/+*^ and *Scn11a*^*+/L799P*^ mice. Fig 2B displays on the left side a DRG section in which several neurons were labeled with the anti-CGRP antibody (see stars). When the anti-CGRP antibody was omitted, no neurons were labeled (Fig 2B, DRG section on the right). Fig 2C shows how the proportions of labeled neurons were determined. Each neuron was identified by radius and grey value. The left panel displays the grey value of all neurons in the absence of the anti-CGRP antibody (background grey). Neurons from *Scn11a*^*+/+*^ and *Scn11a*^*+/L799P*^ mice which showed grey values higher than the background value (panels 2 and 3 in Fig 2C), were considered CGRP-positive.

We measured the release of CGRP from isolated sciatic nerves. The protocol is shown in Fig 2D. After measuring basal release of CGRP, the nerve was exposed to capsaicin for 5 minutes. Capsaicin evoked the release of CGRP from sciatic nerve axons in a concentration-dependent manner (Fig 2E). The comparison of the CGRP release evoked by capsaicin 10–7 M from nerves of Scn11a+/+ and Scn11a+/L799P mice (3–7 months) revealed that the release of CGRP was strongly decreased from sciatic nerve axons of Scn11a+/L799P mice (Fig 2F). An age-dependent effect could not be observed in these experiments.

### Measurement of gastrointestinal transit time and peristaltic

**Fig 3:**
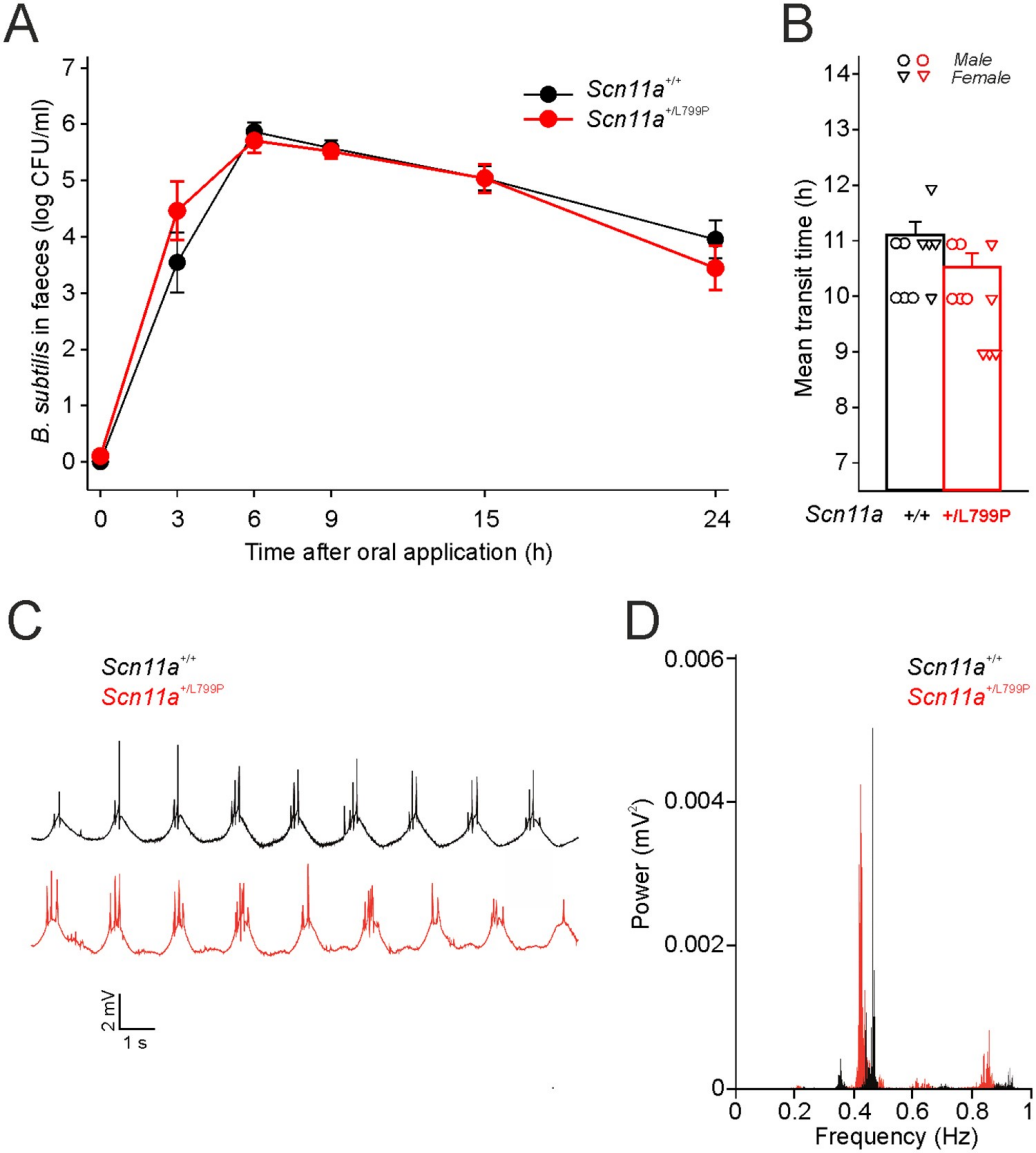
Gastrointestinal transit time (GITT) and spontaneous mechanical activity of the intestine. No significant differences between both genotypes were observed regarding GITT at individual time points of the experiment (A) and for the mean of GITT (B) (n = 10 per group, 4–7 months old). Representative field potential recordings (C) and power spectra of field potential recordings (D) of the Tunica muscularis of the small intestine indicate a slight shift towards less frequent spontaneous peristaltic in *Scn11a*^*+/L799P*^ mice (n = 3 per group). Values are mean ± SEM. *CFU* colony forming unit.

Gastrointestinal transit time (GITT) was monitored over a time span of 24 h after force-feeding with an inert transit marker (spores of *Bacillus subtilis*). Feces were collected after 3, 6, 9, 15 and 24 h and GITT was calculated for each time point. We did neither observe significant differences in GITT at individual time points (Fig 3A), nor in the mean (Fig 3B) between both genotypes of mature mice (4–7 months old). Because *Scn11a*^*+/L799P*^ mice showed a slight decrease in the mean GITT we also analyzed intestinal peristaltic ex vivo. Representative field potential recordings (Fig 3C) and power spectra of field potential recordings (Fig 3D) of isolated segments of the Tunica muscularis of the small intestine indicate a slight shift towards less frequent spontaneous peristaltic events in *Scn11a*^*+/L799P*^ mice.

### Quantification of grip strength and muscle fiber composition

**Fig 4:**
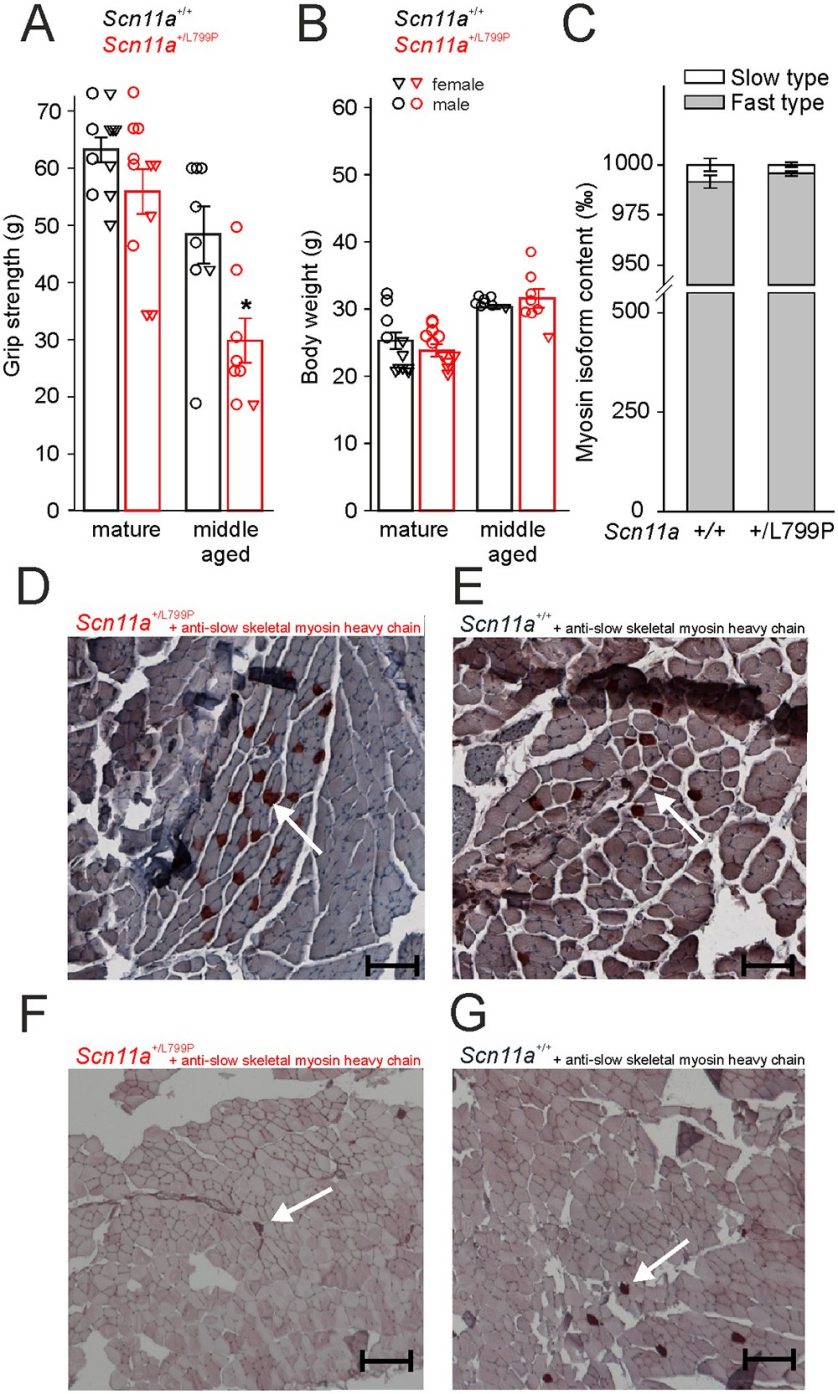
Quantification of grip strength and muscle fiber histology. (A) Middle aged (9–10 months) but not mature adult (3–7 months) *Scn11a*^*+/L799P*^ mice showed a significant decrease in grip strength compared to age-matched *Scn11a*^*+/+*^ littermates (n = 8–11 per group). (B) Both genotypes did not differ in their body weights (n = 8–11 per group). (C) Both genotypes also did not differ in the composition of slow and fast type muscle fibers of the Musculus tibialis cranialis (n = 6 per group; all *Scn11a*^*+/+*^ mice and 4 out of 6 *Scn11a*^*+/L799P*^ used for muscle histology were also used for grip strength testing). (D, E) Representative images of muscle fiber staining using anti-slow skeletal myosin heavy chain antibody of cross sections of the forelimbs of *Scn11a*^*+/L799P*^ mice (D) and *Scn11a*^*+/+*^ mice (E) (bar 200 μm) showing positively labeled slow (type 1) myosin isoform muscle fibers (marked by arrows). (F, G) Muscle fiber staining using anti-slow skeletal myosin heavy chain antibody of cross sections of the Musculus tibialis cranialis of *Scn11a*^*+/L799P*^ mice (F) and *Scn11a*^*+/+*^ mice (G) (bar 200 μm). Values are mean ± SEM. *p < 0.05.

Maximum forelimb grip strength was quantified with single weights in a range between 2.8 and 103 g. We found a significant decrease in grip strength (by ~38%) in middle aged (9–10 months) *Scn11a*^*+/L799P*^ mice compared to *Scn11a*^*+/+*^ mice (Fig 4A). Again, mainly male mice were tested at this time point. Mature adult (3–7 months) mice did not show significant differences between both genotypes. Notably both groups of mice did not differ in their physical constitution shown on the basis of the body weight (Fig 4B). To analyze whether this decrease in grip strength of middle aged *Scn11a*^*+/L799P*^ mice is reflected in an alteration of muscle fiber composition we stained slow (type 1) muscle fibers in cross sections of the forelimb and in the separated Musculus tibialis cranialis. Muscle fiber typing was performed 5 weeks after quantification of grip strength and revealed no significant differences in the content of slow muscle fibers between both genotypes for Musculus tibialis cranialis (Fig 4C). Fig 4D–4G show representative images of fiber staining of muscle fibers with an antibody for slow skeletal myosin heavy chain in which few muscle fibers are labeled. Fig 4D and 4E display areas from the forelimb (for whole section see S1 Fig), Fig 4F and 4G show areas of the Musculus tibialis cranialis.

### Gait analysis and joint diameters

**Fig 5:**
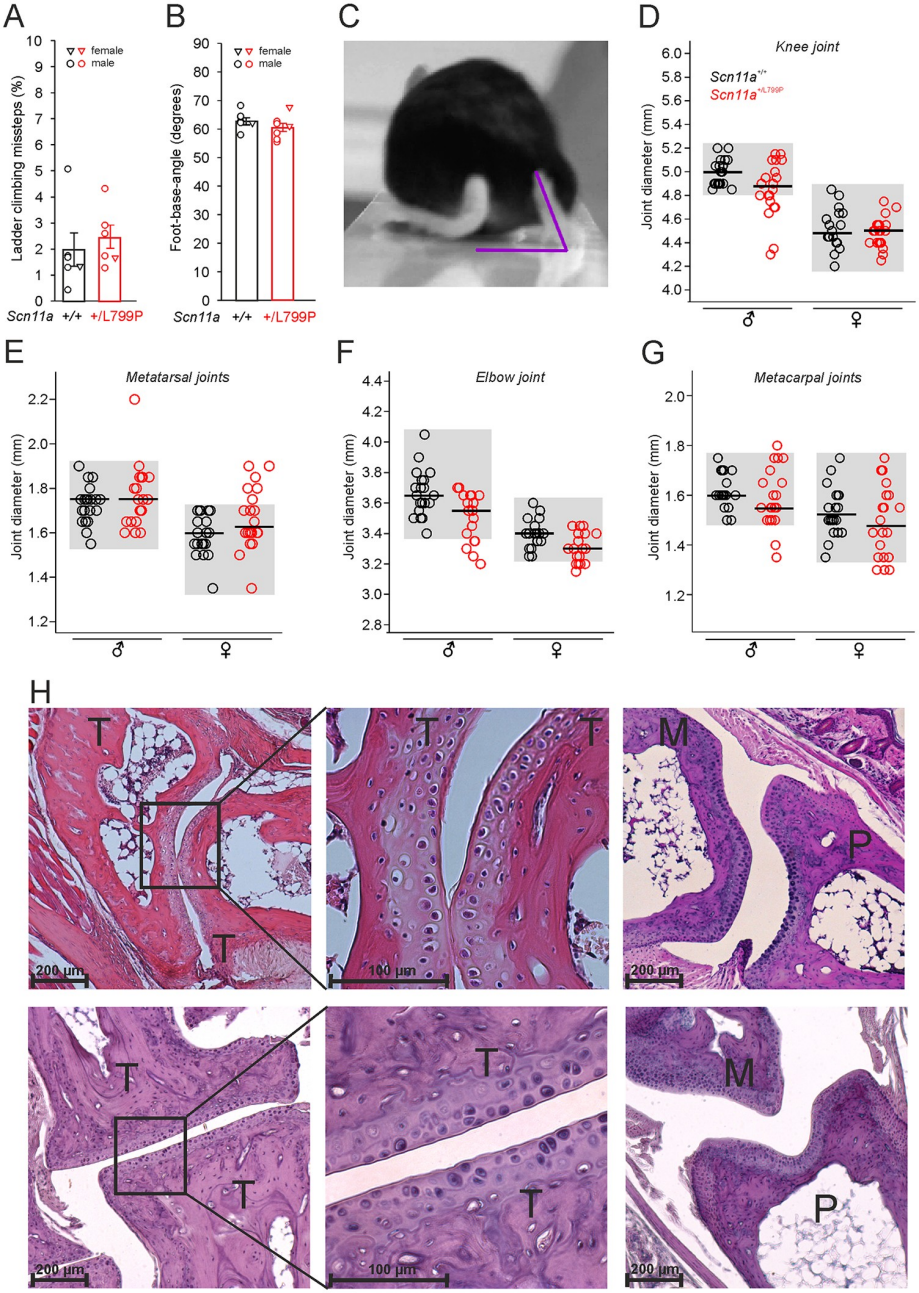
Quantitative gait analysis and joint diameters. No differences in the percentage of ladder climbing missteps (A) and in the foot-base-angle (B) were observed between *Scn11a*^*+/L799P*^ mice and *Scn11a*^*+/+*^ mice (9–10 months, n = 6–8 per group, values are mean ± SEM). Triangles indicate values of individual female mice, cicles indicate values of individual male mice. (C) Representative single frame of a video sequence for visualization of the foot-base-angle (in purple) at toe-off position. Diameters of knee joints (D), metatarsal joints (E), elbow joints (F) and metacarpal joints (G) were compared between both genotypes (sex-matched, 2–6 months, n = 20 per genotype). Horizontal bars indicate the median of each group and grey boxes indicate the range of values from *Scn11a*^*+/+*^ mice for each comparison. (H) Histology after H&E staining of the paws from *Scn11a*^*+/L799P*^ mice with enhanced metatarsal joint diameters (upper panel) and representative images from *Scn11a*^*+/+*^ mice (lower panel). The left microphotographs show metatarsal joints, the middle microphotographs display magnifications of the areas indicated on the left, the right microphotographs show metatarsophalangeal joints. *Scn11a*^*+/L799P*^ mice with enhanced metatarsal joint diameters did not show pathological alterations. ♂ male, ♀ female, M metatarsal bone, T tarsal bone, P first phalanges.

To evaluate motor function two tests were performed. We quantified complex motor behavior („skilled walking“) with the ladder-climbing test and analyzed potential deficits in motor coordination (e.g. ataxia [26]) with the beam-walk balance test. None of these tests revealed a disturbance in muscle function in middle aged (9–10 months) *Scn11a*^*+/L799P*^ mice compared to *Scn11a*^*+/+*^ mice. No significant differences in the proportion of missteps (Fig 5A) and in the foot-base-angle (Fig 5B) were observed between both genotypes. We also did not observe any falls off the beam in both genotypes. Fig 5C displays a representative single frame of a video sequence for visualization of the foot-base-angle (in purple) at toe-off position. Mainly male mice were tested.

We also examined several joints of the forepaws and hind paws to detect joint deformities. The diameters of the knee joints (medio-lateral), of the elbow joints (medio-lateral) and of the metatarsal and metacarpal joints (dorso-plantar) were measured (Fig 5D–5G). Male and female mice were examined separately excluding differences in physical constitution (body weights in G: male Scn11a+/+ 28.2±0.3, female Scn11a+/+ 21.7±0.4, male Scn11a+/L799P 27.5±0.5, female Scn11a+/L799P 22.7±0.3; n = 10 per group). A few Scn11a+/L799P mice showed enhanced diameters of metatarsal joints (7 paws out of 40) and of metacarpal joints (1 paw out of 40) compared to the ranges of joint diameters of Scn11a+/+ mice. Histological evaluation of the affected joints did not indicate pathological alterations, neither regarding inflammatory infiltration nor bone structure (Fig 5H).

## Discussion

This study yielded several salient findings. First, similar as humans with the rare gain-of-function mutation *p*.*Leu811Pro* in *SCN11A*, *Scn11a*^*+/L799P*^ mice show significant changes in several organs with similar manifestations. These findings indicate important Na_v_1.9 channel functions in several organs across different species. Second, the data strongly support the pathophysiological relevance of increased basal activity of Na_v_1.9 channels for (altered) sensory experiences (pain and itch) as well as for some malfunctions of the motor system and of the gastrointestinal tract.

The previous analysis of pain behavior of *Scn11a*^*+/L799P*^ mice had already shown that Na_v_1.9 channels with increased basal activity decrease pain sensitivity which may correspond to congenital insensitivity to pain in the human *p*.*Leu811Pro* mutation [3]. Since the *Scn11a*^*+/L799P*^ mice developed with time also enhanced scratching we conclude that they began to experience pruritus, reminiscent of pruritus in patients with the *p*.*Leu811Pro* mutation [3]. It is noteworthy that this sensory abnormality was not observed in younger, adult mice but only in mice at the middle age (9–12 months). Importantly, scratching was observed before skin lesions became visible, suggesting that skin lesions may develop as a consequence of enhanced scratching (possibly also favored by pain insensitivity). Scratching does not seem to be the expression of a general neurologic disorder because grooming behavior was normal [27].

Our data support that Na_v_ channels are involved in itch sensation. Paroxysmal itch was found in a gain-of-function mutation in *SCN9A* (coding for Na_v_1.7) [28], and in a gain-of-function Na_v_1.8 mutation in *SCN10A* (coding for Na_v_1.8) in painful neuropathy [29]. In Na_V_1.9^-/-^ mice Salvatierra et al. [5] showed a strong reduction in acute scratching to the pruritogens histamine, CQ (a MrgprA3 activator) and BAM8-22 (MrgprC11 activator). Furthermore, their *Na*_*v*_*1*.*9*^*L799P/WT*^ mice generated by using a CRISPR/Cas9 strategy to introduce the mutation into a N-terminal sfGFP (superfolder green fluorescent protein)-tagged Na_v_1.9 mouse line, showed increased spontaneous scratching which was attributed to a hyperexcitability of MrgprA3^+^ neurons [5].

The most interesting observation of enhanced scratching in the *Scn11a*^*+/L799P*^ mice is that it is combined with impaired pain perception. Leipold et al. [3] explained the impaired pain perception in *Scn11a*^*+/L799P*^ mice as the consequence of a process in which increased activation of Na_v_1.9 channels causes a progressive inactivation of neurons. If one assumes that the processing of pain- and itch-evoking stimuli in sensory neurons is similar, it is difficult to understand why hyperactivity of Na_v_1.9 channels and progressive neuronal inactivation causes a reduction of pain perception but an increase of itch perception. In line with Leipold et al. [3], Salvatierra et al. [5] found that most MrgprA3 neurons from *Na*_*v*_*1*.*9*^*L77P/WT*^ mice were unable to fire action potentials even in response to large current injections. But a subset of MrgprA3^+^ neurons with depolarized resting membrane potentials could still fire action potentials in response to small current injections, and they suggested that this small subset of neurons may contribute to spontaneous itch seen in *Na*_*v*_*1*.*9*^*L77P/WT*^ mice and human patients [5]. It seems to be necessary to investigate the encoding of both noxious and pruritogenic stimuli in sensory neurons of *Scn11a*^*+/L799P*^ mice in suitable preparations such as a skin-nerve preparation in order to find out whether both reduced pain perception and enhanced itch sensation can be explained by the functional Na_v_1.9 abnormality in primary afferents or whether complex interactions between different sensory neurons and between sensory neurons and circuits in the central nervous system must be considered important. Compared to wild-type *Scn11a*^*+/+*^ littermates, *Scn11a*^*+/L799P*^ mice do not exhibit obvious morphological alterations of sensory nerves, and they show a regular innervation of the epidermis [3]. Here we show in addition that the proportion of CGRP-positive DRG neurons was not different in *Scn11*^*+/+*^ and *Scn11a*^*+/L799P*^ mice. Their size distribution and grey intensity was similar in both genotypes.

A second salient finding of our study is that nerves from *Scn11a*^*+/L799P*^ mice showed a significant reduction of the capsaicin-evoked release of CGRP although the proportion of CGRP-positive DRG neurons was unaltered in *Scn11a*^*+/L799P*^ mice. CGRP which is released from peptidergic unmyelinated and thinly myelinated sensory neurons upon stimulation, has been implicated in injury, neurogenic inflammation, pain, and itch. CGRP is a potent vasodilator, it is involved in the recruitment of inflammatory cells and synergizes with substance P for mast cell degranulation [8]. CGRP enhances wound healing by direct stimulation of keratinocytes and fibroblasts [9, 10]. Furthermore CGRP knock-out mice exhibited deficient wound closure compared to wild-type controls [20]. CGRP also contributes to activation of sensory neurons. E.g. CGRP enhances TTX-resistant sodium currents in cultured rat dorsal ganglion neurons [12]. CGRP has been implicated in itch because ablation of CGRP-expressing sensory neurons reduced pruritogen-evoked itch [13]. Chronic itch can also occur in the context of neurogenic inflammation [30]. The reduction of the release of CGRP may be strongly involved in some abnormalities observed in *Scn11a*^*+/L799P*^ mice, e.g. slow-healing wounds and reduced pain sensitivity, but it is unlikely, that CGRP is directly involved in the generation of itch because then the CGRP release should be increased. An insufficient activation of calcium ion channels in *Scn11a*^*+/L799P*^ neurons may be the reason for reduced CGRP release [3].

Na_v_ channels are also important in the motor system. Most prominent in skeletal muscle is Na_V_1.4 which carries almost all of the inward Na^+^ currents of action potentials [31]. Both Na_V_1.8 and Na_V_1.9 are expressed in sensory neurons mediating the exercise pressor reflex [32, 33]. In spinal cord motoneurons Na_V_1.9 activity was linked to axon growth and axonal regeneration [34]. The present study shows that *Scn11a*^*+/L799P*^ mice exhibit a significant decrease in grip strength. Compared to *Scn11a*^*+/+*^ mice, the *Scn11a*^*+/L799P*^ mice did not exhibit alterations in muscle fiber composition suggesting that reduction of grip strength is neuronally mediated. Interestingly, the fiber composition of skeletal muscle in mice is substantially different to those in humans. Mouse forelimb muscles are predominantly composed of the fast (type 2) myosin heavy chain isoforms and the slow (type 1) isoform only constitutes <6% [35]. This may preclude direct comparison to humans carrying the gain-of-function mutation *p*.*Leu811Pro* but reduced grip strength may correspond to mild muscle weakness complained by the humans with the Na_V_1.9 variant *p*.*Leu811Pro*. From our histological examination of joints it is unlikely that structural joint abnormalities (observed as Charcot-like arthropathies in humans) contribute to the changes of motor performance.

Finally, we studied gastrointestinal motility because Na_V_1.9 is expressed in myenteric sensory neurons and in neurons of the colonic migrating motor complex (CMMC) [20, 36]. The CMMC is a major pattern of spontaneous contractile activity and Na_V_1.9 null mice exhibit a higher frequency but shorter duration of CMMCs compared to wild-type controls. This phenotype of Na_V_1.9 null mice is connected to an increased excitability of myenteric neurons since Na_V_1.9 regulates cellular depolarization [36]. We did not find significant differences of gastrointestinal transit time (GITT) between *Scn11a*^*+/+*^ and *Scn11a*^*+/L799P*^ mice but measurement of peristalsis in vitro revealed a slight shift towards less frequent spontaneous peristaltic events in *Scn11a*^*+/L799P*^ mice. This may correspond to intestinal dysmotility observed in humans with the Na_V_1.9 variant *p*.*Leu799Pro*.

In summary, *Scn11a*^*+/L799P*^ mice show significant changes similar to those in the rare human *p*.*Leu811Pro* mutation, indicating important Na_v_1.9 channel functions in several organs across different species. The data support the pathophysiological relevance of increased basal activity of Na_v_1.9 channels for sensory abnormalities (pain and itch) and suggest resulting malfunctions of the motor system and of the gastrointestinal tract. *Scn11a*^*+/L799P*^ mice are suitable to investigate the role of Na_v_1.9, and to explore the pathophysiological changes and mechanisms which develop as a consequence of Na_v_1.9 hyperactivity in man.

The present study has several limitations. First, female mice were underrepresented in some experimental groups so that sex differences may have been unrecognized (S1 Table). Since the gain-of-function mutation *p*.*Leu811Pro* in *SCN11A* was observed in male and female patients and since previous studies on the mice with the orthologous mutation *p*.*Leu799Pro* did not separately analyse male and female mice, we did neither plan for sex-specific analysis throughout the study nor select mice regarding sex. Second, not all measurements were performed in middle aged mice. Only during the ongoing study it turned out that some differences develop later in life. This was unexpected since the human mutation shows its phenotype at the young age and since the pain phenotype of *Scn11a*^*+/L799P*^ mice is also present at a young age. For ethical reasons the observation time had to be limited to the life span before severe skin lesions developed.

## Supporting information

S1 Table Comparison of mean gender differences. (PDF)

S1 File Ebbinghaus Scn11a data. (XLSX)

S1 Fig Muscle fiber staining. (DOCX)

S1 Checklist (PDF)
