# Supplementary material for: Gain-of-function mutation in SCN11A causes itch and affects neurogenic inflammation and muscle function in Scn11a+/L799P mice
Source: PLoS One. 2020 Aug 20;15(8):e0237101. doi: 10.1371/journal.pone.0237101 (PMC7440628; doi:10.1371/journal.pone.0237101)

S1 Table. Comparison of mean gender differences.

<table>
<tr><td rowspan="2">Figure
(description; unit)</td><td colspan="2">Scn11a$^{+/+}$ (mean ± SEM)</td><td colspan="2">Scn11a$^{+/L799P}$ (mean ± SEM)</td></tr>
<tr><td>female Scn11a$^{+/+}$ (n)</td><td>male Scn11a$^{+/+}$ (n)</td><td>female Scn11a$^{+/L799P}$ (n)</td><td>male Scn11a$^{+/L799P}$ (n)</td></tr>
<tr><td rowspan="2">1A (Scratching, mature mice; bouts/ 60 min)</td><td colspan="2">4.54 ± 1.31</td><td colspan="2">3.00 ± 0.74</td></tr>
<tr><td>6.86 ± 2.06 (6)</td><td>1.83 ± 0.54 (7)</td><td>1.75 ± 1.11 (4)</td><td>3.56 ± 0.92 (9)</td></tr>
<tr><td rowspan="2">1A (Scratching, middle-aged mice; bouts/60 min)</td><td colspan="2">3.88 ± 1.27</td><td colspan="2">13.86 ± 4.33</td></tr>
<tr><td>7.00 ± 0 (2)</td><td>2.83 ± 1.27 (6)</td><td>6.00 ± 0 (1)</td><td>15.17 ± 4.88 (6)</td></tr>
<tr><td rowspan="2">1B (Grooming, middle-aged mice; chains/60 min)</td><td colspan="2">25.22 ± 2.69</td><td colspan="2">28.13 ± 2.30</td></tr>
<tr><td>30.67 ± 6.12 (3)</td><td>22.50 ± 2.32 (6)</td><td>28.00 ± 1.00 (2)</td><td>28.17 ± 3.13 (6)</td></tr>
<tr><td rowspan="2">2A (CGRP^{+} neurons, middle-aged mice; %)</td><td colspan="2">35.02 ± 0.92</td><td colspan="2">34.26 ± 2.59</td></tr>
<tr><td>35.95 ± 0.87 (4)</td><td>32.50 (1)</td><td>34.95 ± 4.35 (2)</td><td>33.98 ± 3.48 (5)</td></tr>
<tr><td rowspan="2">2E (CGRP release, mature mice; pg/ml)
0 M Capsaicin</td><td colspan="2">25.15 ± 2.61</td><td colspan="2">-</td></tr>
<tr><td>21.40 ± 3.50 (2)</td><td>28.90 ± 0.60 (2)</td><td>-</td><td>-</td></tr>
<tr><td rowspan="2">2E (CGRP release, mature mice; pg/ml)
10^{-8} M Capsaicin</td><td colspan="2">35.33 ± 2.79</td><td colspan="2">-</td></tr>
<tr><td>33.30 ± 5.70 (2)</td><td>37.35 ± 2.45 (2)</td><td>-</td><td>-</td></tr>
</table>

Supplement Table 1 continued

**Figure** (description; unit)	**Scn11a**$^{+/+}$ (mean ± SEM)		**Scn11a**$^{+/L799P}$ (mean ± SEM)	
	**female Scn11a**$^{+/+}$ (n)	**male Scn11a**$^{+/+}$ (n)	**female Scn11a**$^{+/L799P}$ (n)	**male Scn11a**$^{+/L799P}$ (n)
**2E** (CGRP release, **mature** mice; pg/ml) *10^{-7} M Capsaicin*	66.13 ± 8.91		-	
	60.15 ± 13.65 (2)	72.10 ± 14.80 (2)	-	-
**2E** (CGRP release, **mature** mice; pg/ml) *10^{-6} M Capsaicin*	378.8 ± 20.62		-	
	378.8 ± 20.62 (6)	-	-	-
**2F** (CGRP release, **mature** mice; pg/ml) *Baseline (BL) release*	26.60 ± 2.06		11.01 ± 1.11	
	28.13 ± 2.53 (15)	22.77 ± 3.26 (6)	- (0)	28.13 ± 2.53 (15)
**2F** (CGRP release, **mature** mice; pg/ml) *Release after Capsaicin (CAPS)*	49.98 ± 6.16		12.40 ± 2.74	
	48.07 ± 6.96 (7)	53.33 ± 13.14 (4)	- (0)	48.07 ± 6.96 (7)
**2F** (CGRP release, **mature** mice; pg/ml) *Release after Washout*	3.58 ± 1.40		0.05 ± 0.05	
	3.30 ± 2.03 (7)	4.08 ± 1.85 (4)	- (0)	3.30 ± 2.03 (7)
**3A** (B. subtilis in faeces, **mature** mice; log CFU/ ml) *0h after application*	0		0.10 ± 0.10	
	0 (5)	0 (5)	0.20 ± 0.20 (5)	0 (5)

Supplement Table 1 continued

**Figure** (description; unit)	**Scn11a**$^{+/+}$ (mean ± SEM)		**Scn11a**$^{+/L799P}$ (mean ± SEM)	
	**female Scn11a**$^{+/+}$ (n)	**male Scn11a**$^{+/+}$ (n)	**female Scn11a**$^{+/L799P}$ (n)	**male Scn11a**$^{+/L799P}$ (n)
**3A** (B. subtilis in faeces, **mature** mice; log CFU/ ml) *3h after application*	3.54 ± 0.54		4.46 ± 0.52	
	2.79 ± 0.88 (5)	4.29 ± 0.50 (5)	4.82 ± 0.15 (5)	4.10 ± 1.06 (5)
**3A** (B. subtilis in faeces, **mature** mice; log CFU/ ml) *6h after application*	5.87 ± 0.17		5.71 ± 0.21	
	5.99 ± 0.19 (5)	5.75 ± 0.29 (5)	5.74 ± 0.29 (5)	5.67 ± 0.34 (5)
**3A** (B. subtilis in faeces, **mature** mice; log CFU/ ml) *9h after application*	5.57 ± 0.14		5.52 ± 0.13	
	5.77 ± 0.13 (5)	5.38 ± 0.23 (5)	5.49 ± 0.21 (5)	5.55 ± 0.17 (5)
**3A** (B. subtilis in faeces, **mature** mice; log CFU/ ml) *15h after application*	5.04 ± 0.22		5.04 ± 0.25	
	5.02 ± 0.32 (5)	5.06 ± 0.33 (5)	4.85 ± 0.39 (5)	5.23 ± 0.47 (5)
**3A** (B. subtilis in faeces, **mature** mice; log CFU/ ml) *24h after application*	3.95 ± 0.34		3.44 ± 0.39	
	3.93 ± 0.47 (5)	3.97 ± 0.54 (5)	3.16 ± 0.65 (5)	3.72 ± 0.47 (5)
**3B** (Mean transit time, **mature** mice; h)	11.10 ± 0.24		10.53 ± 0.25	
	11.33 ± 0.42 (5)	10.88 ± 0.23 (5)	10.10 ± 0.39 (5)	10.95 ± 0.22 (5)

Supplement Table 1 continued

**Figure** (description; unit)	**Scn11a**$^{+/+}$ (mean ± SEM)		**Scn11a**$^{+/L799P}$ (mean ± SEM)	
	**female Scn11a**$^{+/+}$ (n)	**male Scn11a**$^{+/+}$ (n)	**female Scn11a**$^{+/L799P}$ (n)	**male Scn11a**$^{+/L799P}$ (n)
**4A** (Grip strength, **mature** mice; g)	63.19 ± 2.24		55.93 ± 3.90	
	62.67 ± 3.00 (7)	64.10 ± 3.79 (4)	48.13 ± 5.92 (5)	62.43 ± 3.72 (6)
**4A** (Grip strength, **middle-aged** mice; g)	48.21 ± 5.00		29.68 ± 3.93	
	42.10 (1)	49.09 ± 5.68 (7)	19.00 (1)	31.21 ± 4.18 (7)
**4B** (Body weight, **mature** mice; g)	25.04 ± 1.16		24.55 ± 0.88	
	22.71 ± 0.63 (7)	29.13 ± 1.48 (4)	21.80 ± 0.58 (5)	26.83 ± 0.57 (6)
**4B** (Body weight, **middle-aged** mice; g)	30.38 ± 0.17		31.36 ± 1.36	
	30.30 (1)	30.39 ± 0.20 (7)	25.70 (1)	32.17 ± 1.26 (7)
**4C** (Slow type myosin isoform, **middle-aged** mice; ‰)	8.47 ± 3.13		4.31 ± 1.16	
	22.73 (1)	5.62 ± 1.59 (5)	3.44 ± 1.51 (3)	5.19 ± 1.93 (3)
**4C** (Fast type myosin isoform, **middle-aged** mice; ‰)	991.53 ± 3.13		995.69 ± 1.16	
	977.27 (1)	994.38 ± 1.59 (5)	996.56 ± 1.51 (3)	994.81 ± 1.93 (3)

Supplement Table 1 continued

**Figure** (description; unit)	**Scn11a**$^{+/+}$ (mean ± SEM)		**Scn11a**$^{+/L799P}$ (mean ± SEM)	
	**female Scn11a**$^{+/+}$ (n)	**male Scn11a**$^{+/+}$ (n)	**female Scn11a**$^{+/L799P}$ (n)	**male Scn11a**$^{+/L799P}$ (n)
**5A** (Ladder climbing missteps, **middle-aged** mice; %)	1.94 ± 0.66		2.44 ± 0.46	
	1.34 (1)	2.06 ± 0.80 (5)	1.69 (1)	2.59 ± 0.53 (5)
**5B** (Foot-base-angle, **middle-aged** mice; °)	62.68 ± 1.15		60.55 ± 1.37	
	61.80 (1)	62.83 ± 1.35 (6)	64.11 ± 3.27 (2)	59.36 ± 1.30 (6)
**5D** (Knee joint diameter, **mature** mice; mm)	4.75 ± 0.04		4.67 ± 0.04	
	4.51 ± 0.04 (20)	4.99 ± 0.02 (20)	4.49 ± 0.03 (20)	4.85 ± 0.05 (20)
**5E** (Metatarsal joint diameter, **mature** mice; mm)	1.66 ± 0.02		1.71 ± 0.02	
	1.60 ± 0.02 (20)	1.73 ± 0.02 (20)	1.67 ± 0.03 (20)	1.76 ± 0.03 (20)
**5F** (Elbow joint diameter, **mature** mice; mm)	3.53 ± 0.03		3.41 ± 0.03	
	3.40 ± 0.02 (20)	3.67 ± 0.03	3.30 ± 0.02 (20)	3.52 ± 0.04 (20)
**5G** (Metacarpal joint diameter, **mature** mice; mm)	1.58 ± 0.01		1.54 ± 0.02	
	1.54 ± 0.02 (20)	1.62 ± 0.02 (20)	1.49 ± 0.03 (20)	1.59 ± 0.03 (20)

Supplement Table 1 continued

Power spectra of field potential recordings of the Tunica muscularis of the small intestine as shown in Figure 3D. (A) Both genotypes and all genders included are displayed. (B) Power spectra of *Scn11a*$^{+/+}$ mice are divided for male and female mice. *m* male, *f* female

A

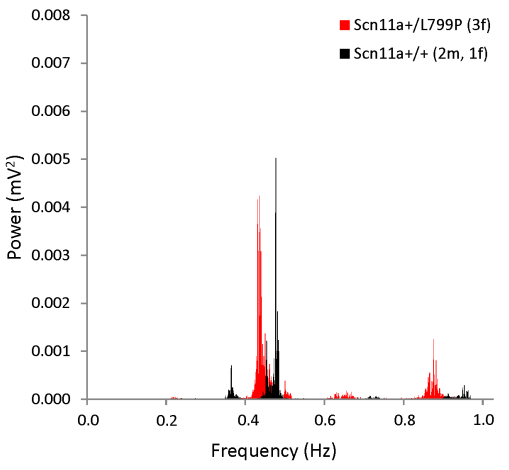

B

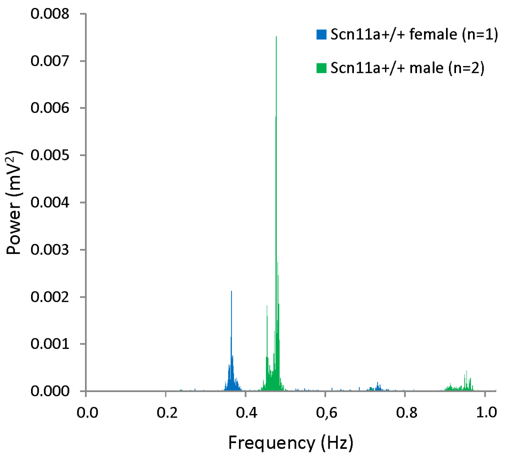

Supplement: S1 Table — (PDF) [file pone.0237101.s001.pdf]
