# Supplementary material for: Gain-of-function mutation in SCN11A causes itch and affects neurogenic inflammation and muscle function in Scn11a+/L799P mice
Source: PLoS One. 2020 Aug 20;15(8):e0237101. doi: 10.1371/journal.pone.0237101 (PMC7440628; doi:10.1371/journal.pone.0237101)

**S1 Fig**

Muscle fiber staining. Representative images of muscle fiber staining using anti-slow skeletal myosin heavy chain antibody of cross sections of the forelimbs of *Scn11a^+/L799P^* mice (A) and *Scn11a^+/+^* mice (B) (magnification 10x, bar 500 µm). The marked squares are displayed in Figure 4D and Figure 4E.


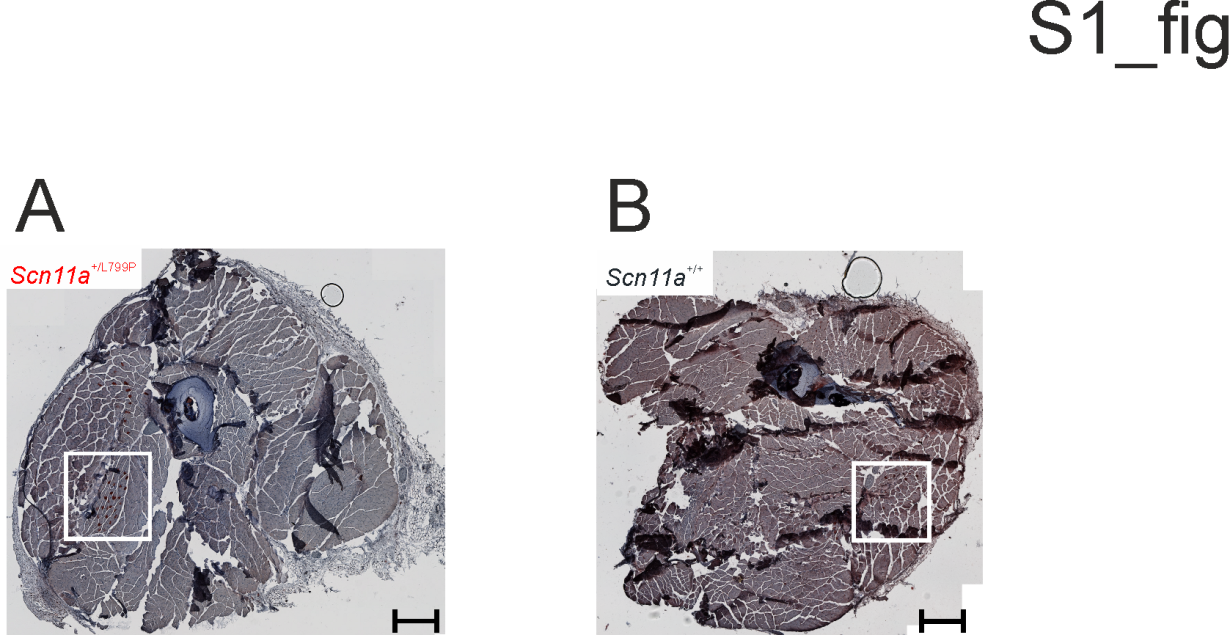

Supplement: S1 Fig — (DOCX) [file pone.0237101.s003.docx]
